# Differential Gene Expression in Colon Tissue Associated With Diet, Lifestyle, and Related Oxidative Stress

**DOI:** 10.1371/journal.pone.0134406

**Published:** 2015-07-31

**Authors:** Martha L. Slattery, Daniel F. Pellatt, Lila E. Mullany, Roger K. Wolff

**Affiliations:** Department of Internal Medicine, University of Utah School of Medicine, Salt Lake City, Utah, United States of America; University of Saarland Medical School, GERMANY

## Abstract

Several diet and lifestyle factors may impact health by influencing oxidative stress levels. We hypothesize that level of cigarette smoking, alcohol, anti-inflammatory drugs, and diet alter gene expression. We analyzed RNA-seq data from 144 colon cancer patients who had information on recent cigarette smoking, recent alcohol consumption, diet, and recent aspirin/non-steroidal anti-inflammatory use. Using a false discovery rate of 0.1, we evaluated gene differential expression between high and low levels of exposure using DESeq2. Ingenuity Pathway Analysis (IPA) was used to determine networks associated with de-regulated genes in our data. We identified 46 deregulated genes associated with recent cigarette use; these genes enriched causal networks regulated by *TEK* and *MAP2K3*. Different differentially expressed genes were associated with type of alcohol intake; five genes were associated with total alcohol, six were associated with beer intake, six were associated with wine intake, and four were associated with liquor consumption. Recent use of aspirin and/or ibuprofen was associated with differential expression of TMC06, ST8SIA4, and STEAP3 while a summary oxidative balance score (OBS) was associated with SYCP3, HDX, and NRG4 (all up-regulated with greater oxidative balance). Of the dietary antioxidants and carotenoids evaluated only intake of beta carotene (1 gene), Lutein/Zeaxanthine (5 genes), and Vitamin E (4 genes) were associated with differential gene expression. There were similarities in biological function of de-regulated genes associated with various dietary and lifestyle factors. Our data support the hypothesis that diet and lifestyle factors associated with oxidative stress can alter gene expression. However genes altered were unique to type of alcohol and type of antioxidant. Because of potential differences in associations observed between platforms these findings need replication in other populations.

## Introduction

Diet and lifestyle factors have been associated with several chronic diseases including colon cancer. Several of these diet and lifestyle factors may influence risk through their impact on inflammation and oxidative stress. Cigarette smoking has been associated with colon cancer and other chronic disease and has been linked to inflammation through increased oxidative stress [1–8]. Likewise, alcohol intake has been associated with risk of colon cancer and may act through several mechanisms, including increasing levels of oxidative stress [9–12]. One of the factors most consistently inversely associated with colon cancer risk is use of aspirin and/or non-steroidal anti-inflammatory drugs (NSAIDs). Dietary antioxidants likewise have been associated with colon cancer risk [13–16]. We and others have shown that the oxidative balance score (OBS) is associated with colon and rectal cancer [17–19], with risk lowest among those with a score that is higher in antioxidants and low in pro-oxidant factors. Components of this score are cigarette smoking, use of aspirin or non-steroidal anti-inflammatory drugs (NSAIDs), and dietary pro- and anti-oxidants. While it has been hypothesized that these factors act as anti-oxidants or pro-oxidants, it is unknown if they directly influence gene expression that may alter these and other pathways. However, changes in gene expression were observed among men in an intervention study of diet and lifestyle modification [20], supporting the hypothesis that diet and lifestyle factors influence gene expression. Others also have suggested that oxidative stress may modulate gene expression [21].

In this study we examine how dietary antioxidants, cigarette smoking, alcohol consumption, NSAID use, and the OBS influence gene expression in non-tumor colonic tissue. We hypothesize that differences in gene expression levels with be associated with level of diet and lifestyle factors that influence inflammation and oxidative stress. We hope that information on how these diet and lifestyle factors alter gene expression will provide direct insight into how they influence cancer and other chronic diseases.

## Methods

Total RNA was available from colonic non-tumor tissue for 175 colon cancer cases who were part of the Diet, Activity, and Lifestyle study, an incident, population-based, case-control study of colon cancer from Utah and the Kaiser Permanente Medical Research Program (KPMRP), and the Twin Cities Metropolitan area. Cases had tumor registry verification of a first primary adenocarcinoma of the colon and were diagnosed between October 1991 and September 1994. Tumor tissue blocks were obtained for 97% of all Utah cases and for 85% of all KPMRP cases [22] and included those who signed informed consent and those retrieved by local tumor registries and sent to study investigators without personal identifiers. Individuals with known adenomatous polyposis coli (APC), Crohn’s disease, or inflammatory bowel disease were not eligible for the study. Individuals with MSI high tumors were sequenced for inherited mutations in mismatch repair genes and excluded from the study if such mutations existed [23]. The study was approved by the Institutional Review Board of the University of Utah and at KPMRP. In this study we use colonic non-tumor tissue that was from the same location as the tumor to evaluate how recent cigarette smoking, recent alcohol use, recent use of aspirin or non-steroidal anti-inflammatory agents (NSAIDs), and recent dietary intake of antioxidants influence gene expression.

### Diet and Lifestyle Data

Data were collected by trained and certified interviewers using laptop computers. All interviews were audio-taped as previously described and reviewed for quality control purposes [24]. The referent period for the study was two years prior to diagnosis for cases and selection for controls. Dietary information was obtained for the referent year from an extensive diet history questionnaire adapted from the validated CARDIA diet history [25]. As part of the study questionnaire, information was collected on regular use and current use of aspirin and non-steroidal anti-inflammatory drugs and cigarette smoking history including start and stop dates for smoking. Alcohol intake of beer, wine, and hard liquor was ascertained for the referent year as well as for 10 and 20 years prior to the referent year.

### RNA processing

RNA was extracted from formalin-fixed paraffin embedded tissues. We assessed slides and tumor blocks that were prepared over the duration of the study prior to the time of RNA isolation to determine their suitability. Older slides produced comparable RNA quality as more recent slides and was not correlated with time lapse between slide preparation and RNA preparation. The study pathologist reviewed slides to delineate tumor and non-tumor tissue. Cells were dissected from 1–4 sequential sections on aniline blue stained slides using an H&E slide for reference. Total RNA was extracted, isolated, and purified using the RecoverAll Total Nucleic Acid isolation kit (Ambion), RNA yields were determined using a NanoDrop spectrophotometer.

### Sequencing Library Preparation

Library construction was performed using the Illumina TruSeq Stranded Total RNA Sample Preparation Kit with Ribo-Zero. Briefly, Ribosomal RNA was removed from 100 ng total RNA using biotinylated Ribo-Zero oligos attached to magnetic beads that are complimentary to cytoplasmic rRNA. Following purification, the rRNA-depleted sample is fragmented with divalent cations under elevated temperatures and primed with random hexamers in preparation for cDNA synthesis. First strand reverse transcription is accomplished using Superscript II Reverse Transcriptase (Invitrogen). Second strand cDNA synthesis is accomplished using DNA polymerase I and Rnase H under conditions in which dUTP is substituted for dTTP, yielding blunt-ended cDNA fragments in which the second strand contains dUTP. An A-base is added to the blunt ends as a means to prepare the cDNA fragments for adapter ligation and block concatamer formation during the ligation step. Adapters containing a T-base overhang were ligated to the A-tailed DNA fragments. Ligated fragments were PCR-amplified (13 cycles) under conditions in which the PCR reaction enables amplification of the first strand cDNA product, whereas attempted amplification of the second strand product stalls at dUTP bases and therefore is not represented in the amplified library. The PCR-amplified library was purified using Agencourt AMPure XP beads (Beckman Coulter Genomics). The concentration of the amplified library was measured with a NanoDrop spectrophotometer and an aliquot of the library is resolved on an Agilent 2200 Tape Station to define the size distribution of the sequencing library.

### Sequencing and Data Processing

Sequencing libraries (18 pM) were chemically denatured and applied to an Illumina TruSeq v3 single read flow cell using an Illumina cBot. Hybridized molecules were clonally amplified and annealed to sequencing primers with reagents from an Illumina TruSeq SR Cluster Kit v3-cBot-HS. Following transfer of the flowcell to an Illumina HiSeq instrument, a 50 cycle single-read sequence run was performed using TruSeq SBS v3 sequencing reagents. The single-end 50-base reads from the Illumina HiSeq2500 were aligned to a sequence database containing the human genome (build GRCh37 / hg19, February 2009, from genome.ucsc.edu) plus all splice junctions generated using the USeq MakeTranscriptome application (version 8.8.1, available here: http://useq.sourceforge.net/). Alignment was performed using novoalign version 2.08.01 available from novocraft.com, which also trimmed any adapter sequence. Following alignment, genome alignments to splice junctions were translated back to genomic coordinates using the USeq SamTranscriptomeParser application. The resulting alignments were sorted and indexed using the Picard SortSam application (version 1.100, available here: http://broadinstitute.github.io/picard/). Aligned read counts for each gene were calculated using pysam (https://code.google.com/p/pysam/) and samtools (http://samtools.sourceforge.net/). A python script using the pysam library was given a list of the genome coordinates for each gene, and counts to the exons and UTRs of those genes were calculated. Gene coordinates were downloaded from http://genome.ucsc.edu.

We compared our data to a gene table with 51,041 molecular features. We dropped features that were not expressed in our data or for which the expression was unavailable for the majority of samples. Using the BioMart tool on the Ensembl website (http://www.ensembl.org), we created a list of known regions linked to protein-coding genes from the human GRCh38 gene annotation dataset. Non-protein coding genes were also dropped from our analysis. Our final analysis included 17,462 protein-coding features.

### Statistical Methods

Of the 197 initial tumor/non-tumor tissue pairs, five subjects failed quality control (QC) based on low number of sequence counts for both tumor and non-tumor tissue, and 17 were dropped because the non-tumor colonic tissue failed QC, leaving 175 subjects with high quality expression data. Of these 144 had questionnaire data for diet and lifestyle data for inclusion in the analysis. In terms of specific dietary factors, we focused on carotenoids and antioxidant nutrients, including beta carotene, vitamin C, total alpha tocopherol (vitamin E), lycopene, and lutein + zeaxanthin. We also considered alcohol consumption, recent use of NSAIDs, and current smoking. For each dietary and lifestyle factor, our analysis centered on contrasting gene expression levels of individuals with lower intake or exposure levels to those of individuals with higher intake or exposure levels. Each individual’s intake or exposure was assigned a category based on their dietary and lifestyle data. Dietary data were categorized into tertiles [i.e. low (T1), moderate (T2), or high (T3)] based on the empirical distributions in the population. Cigarette smoking was categorized as never, former, or current smoker. Alcohol was categorized into non-drinker, low intake, or high intake for each type of alcohol. Use of NSAIDs (which included aspirin and/or non-steroidal anti-inflammatory drugs) was categorized as either being a recent user (i.e. using NSAIDs during the referent period) or a non-user. To summarize risk associated with multiple exposures, we developed an oxidative balance score (OBS) that consisted of 13 diet and lifestyle factors that were pro-oxidants (dietary iron and polyunsaturated fat and cigarette smoking) and anti-oxidants (vitamin C, vitamin E, selenium, beta carotene, lycopene, lutein+zeaxanthin, vitamin D, calcium, and folic acid and NSAID use) [17]. To create the OBS, these diet and lifestyle factors were assigned values of 2 for low levels of exposure for each pro-oxidants or high exposure to anti-oxidants (low-risk), one for intermediate levels of exposure, and zero for high levels of exposure to pro-oxidants and low exposure to anti-oxidants (high-risk). The individual scores for the 13 variables were then combined to obtain the OBS. Higher summary score corresponded to greater oxidative balance; individual’s OBSs were categorized as low, intermediate, or high based on tertiles associated with the empirical distribution of the OBSs.

For each variable of interest (specific dietary factors, NSAIDS use, smoking and OBS), we assessed which genes displayed statistically significant differential expression between low and high categories using the Bioconductor package DESeq2 written for the R statistical programming environment. DESeq2 assumes the RNA-seq counts are distributed according to negative binomial distributions. It utilizes generalized linear modeling to test individual null hypotheses of zero log2 fold changes between high and low categories (i.e. no differential expression) for each gene and it employs both an independent-filtering method and the Benjamini and Hochberg [26] procedure to improve power and control the false discovery rate (FDR). For further details regarding DESeq2, see Love et al. [27]. In identifying genes with significant differential expression, an FDR of 0.10 was used.

To help describe the data, we report the average DESeq2-adjusted gene expression levels (adjusted counts) among individuals in the high and low categories of the dietary or lifestyle variables of interest for each differentially expressed gene and include fold change calculations associated with these genes. Included as a descriptive detail rather than reflecting direct DESeq2 output, fold change was calculated as the ratio of a gene’s mean expression among individuals in the high category of a dietary or lifestyle variable to its mean expression among individuals in the low category; a fold change greater than one indicates a positive differential expression (i.e. up-regulated) while a fold change between zero and one indicates a negative differential expression (i.e. down-regulated).

To visualize differential gene expressions between individuals in high and low categories of related diet and lifestyle variable groups, we created heat maps. Each heat map features the log2 transformation of the fold changes, calculated as described above, associated with genes identified as significantly differentially expressed between high and low categories of the diet and lifestyle variables considered for the specific heat map. Our heat maps were created using the heatmap.2 program in the ‘gplots’ package of R (http://cran.r-project.org). Distance between two vectors of log2 transformed fold changes was measured via the Euclidean metric and median linkage was selected for this programs’ agglomerative hierarchical clustering algorithm.

Bioinformatics analysis was performed on the list of Ensemble IDs associated with genes identified as differentially expressed with QIAGEN’s Ingenuity Pathway Analysis (IPA) [28]. We used genes from Ingenuity Knowledge Base and considered both indirect and direct relationships. Networks were limited to 35 molecules and 25 networks per analysis and included both causal and interaction networks. We included all data sources in our IPA assessment, but did not restrict to species or specific tissue when compiling networks. We applied the Benjamini-Hochberg (B-H) multiple testing correction to assess pathways in IPA.

## Results

The majority of study participants were male (Table 1). A large percentage of people never drank alcohol and were not current smokers. The mean age of the population was 64.5 years and 37.3% of the population reported recent use of NSAIDs.

**Table 1:** Description of study population.

		Overall	
		N	%
Sex			
	Male	80	55.6%
	Female	64	44.4%
Smoking Status			
	Never	60	41.7%
	Former	65	45.1%
	Current	19	13.2%
Recent NSAID Use			
	No	89	62.7%
	Yes	53	37.3%
Alcohol Long Term and Current (gm/day)			
	Low	53	36.8%
	Mod	68	47.2%
	High	23	16.0%
Beer Long Term & Current (gm/day)			
	Low	88	61.1%
	Mod	38	26.4%
	High	18	12.5%
Wine Long Term & Current (gm/day)			
	Low	74	51.4%
	Mod	47	32.6%
	High	23	16.0%
Liquor Long Term & Current (gm/day)			
	Low	76	52.8%
	Mod	44	30.6%
	High	24	16.7%
		Median	Inter-quartile Range
Beta carotene (mcg/1000 KCAL)			
	Low^1^	912.3	(668.7, 1058.6)
	Intermediate	1882.1	(1550.4, 2055.5)
	High	3287.4	(2647.2, 4583.7)
Lutein + Zeaxanthin (mcg/1000 KCAL)			
	Low	622.9	(530.2, 745.9)
	Intermediate	1014.3	(918.7, 1081.7)
	High	1650.0	(1388.6, 2172.1)
Lycopene (mcg/1000 KCAL)			
	Low	1424.5	(1075.8, 1846.6)
	Intermediate	2809.5	(2428.2, 3133.7)
	High	4723.0	(3994.0, 5891.7)
Total Alpha Tocopherol (mg/1000 KCAL)			
	Low	3.1	(2.8, 3.3)
	Intermediate	4.1	(4.0, 4.4)
	High	5.4	(5.0, 6.4)
Vitamin C (mg/1000 KCAL)			
	Low	37.0	(32.2, 44.7)
	Intermediate	61.6	(56.1, 65.8)
	High	109.4	(88.5, 132.2)
Oxidative Balance Score			
	Low	10.0	(8.8, 11.0)
	Intermediate	14.0	(13.0, 14.8)
	High	17.5	(16.0, 19.0)

^1^Categories are based on tertiles of the population of approximately 48 people per category

**Fig 1:**
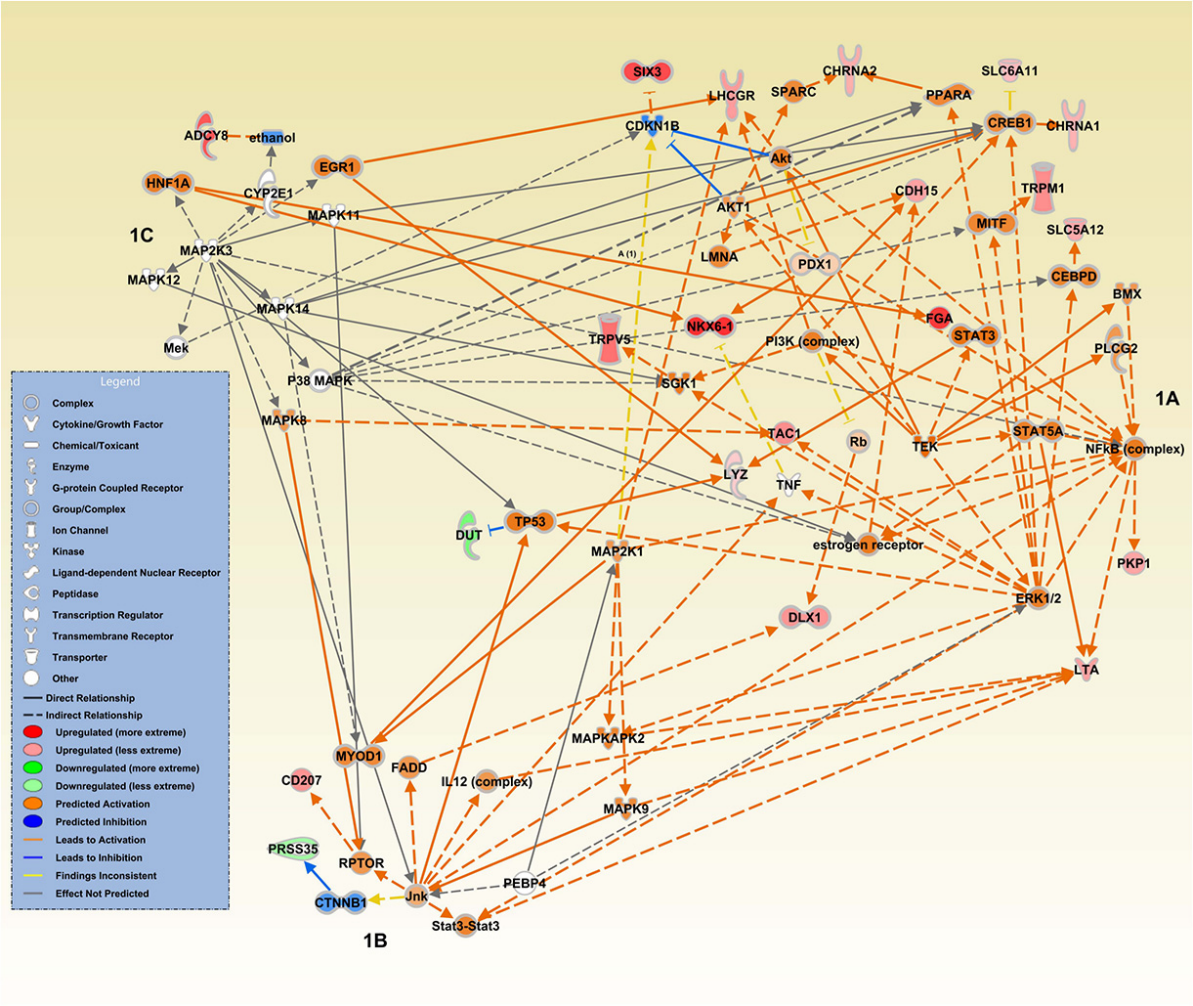
Causal Networks identified by IPA where differentially expressed genes were enriched by recent cigarette smoking. Fig 1A. *TEK* causal network. Fig 1B. *PEBP4* causal network. Fig 1C. *MAP2K3* causal network.

Forty-six genes were differentially expressed between current smokers and non-smokers (S1 Table); the majority of these genes were up-regulated among current smokers. These genes enriched three networks as indicated by the focus molecules (those that are deregulated in our data) and were involved in cell morphology, cellular development, endocrine system development and function, cellular functions and maintenance, lipid metabolism, and vitamin and mineral metabolism (Table 2). Upstream regulators to which significant number of our deregulated genes among smokers were linked included MAPkinase genes *TEK* and *MAP2K3* and *PEBP4* (Fig 1A–1C).

**Table 2:** Causal Networks associated with recent cigarette smoking.

Network	Molecules in Network^1^	Score	Focus Molecules	Top Diseases and Functions
1	3',5'-cyclic-GMP phosphodiesterase, ***C11orf16*, *C1orf61***, CCND1, **CD207, CHRNA2**, CSF2, D-glucose, EWSR1, F13A1, **FGA**, GLIS3, guanosine triphosphate, Hmgn3, INSM1, **LAMB4,** MAFA, MITF, **NKX6-1,** NR4A3, PDGF BB, PLEC, PPARA, PRSS35, **SAG, SIX3**, SRPX, TGFB1, TLE4, TPSAB1/TPSB2, **TRPM1, TRPV5**, VGF, WFS1, **YY2**	30	13	Cell Morphology, Cellular Development, Endocrine System Development and Function
2	**ADCY8,** CEBPD, **CHRNA1,** ECE1, FGF23, GFRA1, GPIIB-IIIA, HTR7, Igha, IL1, KCNK3, **LHCGR, LTA**, Lymphotoxin, **LYZ,** Madcam1, N-arachidonoyl-dopamine, NFkB (complex),NLR,NLRC4,**NLRP4,**Pka,Pkc(s),**PKP1,**PLC,SLC5A12,TAC1,TAC4,TACR2,TACR3,**TECTB**,thyroid hormone,TRPA1,**TRPV5**,UMOD	24	11	Cellular Growth and Proliferation, Organismal Development, Cellular Function and Maintenance
3	ARVCF, BARX1, **CACNG8, CDH15**, CENPB, **CFAP61**, CRELD2, DHCR7, DLG4, **DLX1**, DUT, **FBN3**, FDFT1, GADD45A, GRIA3, HCN1, HELZ, HMGCR, IDI1, IGF2, KCNA3, LSS, MGEA5, MVK, PEX10, **PEX5L,** PMVK, RAB8B, **SLC6A11,** SPATA2, UBC, VSIG1, WDR87, ZDHHC3, ZDHHC7	24	11	Lipid Metabolism, Small Molecule Biochemistry, Vitamin and Mineral Metabolism

^1^Genes in bold were upregulated in our data while genes underlined were down-regulated in our dataset

**Fig 2:**
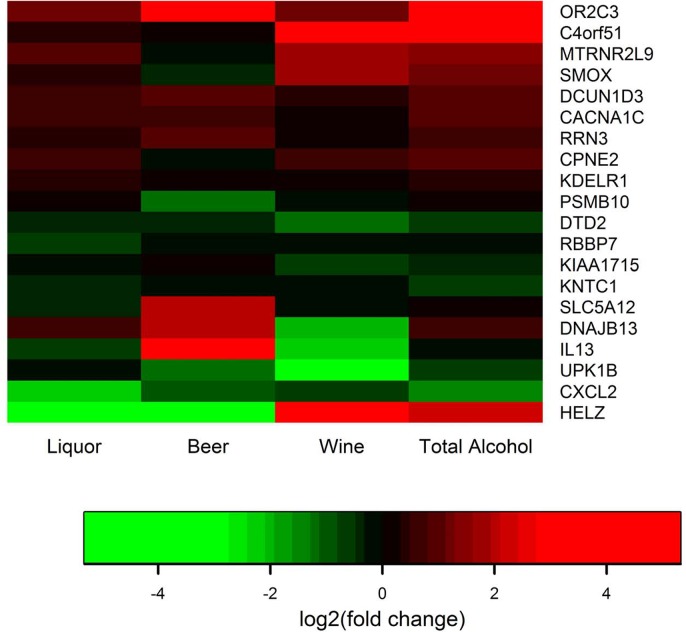
HEATMAP of differentially expressed genes by type of alcohol intake.

Five genes were differentially expressed between high recent alcohol consumers and non-consumers of alcohol (Table 3). Six genes were differentially expressed for high beer consumers versus non-consumers, six genes were differentially expressed for high consumers of wine, and four genes were differentially expressed among liquor consumers versus non-consumers. Both up- and down-regulated genes were observed, although overall alcohol consumption and beer consumption were more likely to be associated with upregulated genes and liquor consumption was more likely to be associated with down-regulated genes. As illustrated in Fig 2, there were uniquely de-regulated genes based on specific type of alcohol consumption (p values corresponding to de-regulated genes by alcohol type can be found in Table 3).

**Table 3:** Associations between alcohol and gene expression.

Gene	Average Adjusted Count, Low Tertile Group	Average Adjusted Count, High Tertile Group	Fold Change	P Value^1^	Q Value^2^	Function/known pathways or disease relationships
**Total Alcohol**						
*KDELR1*	51.32	71.20	1.39	3.63E-05	0.076	Induces phyphorylation of p38 MAP kinases and may impact outcome of ER stress response
*CPNE2*	23.02	42.18	1.83	7.55E-05	0.092	Encodes a calcium dependent protein; regulation of tumor necrosis factor
*CACNA1C*	27.52	50.72	1.84	4.28E-05	0.076	Cellular calcium ion homeostasis; cell-to-cell sginaling; regulation of hormone levels such as insulin; mood disorders and biopolar disease
*KNTC1*	54.14	34.24	0.63	5.00E-05	0.076	Regulation of cell cycle process; personality disorders; diabetes
*HELZ*	3579.58	19213.39	5.37	3.82E-06	0.023	Alter biologic activity of RNA; mediates MAPK signaling
**Beer**						
*RRN3*	20.13	38.32	1.90	1.48E-06	0.017	Required for efficient transcription inition by RNA polymerase I; ERKA and AKT activation (mTOR- signaling pathway
*IL13*	0.28	3.13	11.18	2.84E-05	0.083	Inflammatory response; this cytokine isdown regulates macrophage activity thereby inhibits the production of pro-inflammatory cytokines and chemokines. Involved in ERK signaling and AKT signaling PAK Pathway; asthma
*DNAJB13*	0.85	3.44	4.04	1.90E-05	0.067	Apoptosis; protein folding; gamete generation;
*DCUN1D3*	5.64	11.84	2.10	1.10E-05	0.048	Negative regulation of cell growth; positive regulation of apoptotic process; response to radiation
*OR2C3*	0.08	3.16	39.85	1.99E-06	0.017	Cell surface receptor linked signal transduction; neurological system process; cognition; Olfactory Signaling Pathway; Signaling by G protein coupled receptors (GPCR)
*PSMB10*	15.16	6.96	0.46	7.25E-06	0.042	Endopeptidase activity; immune response; metabolioc processes; cell cycle, mitotic pathways
**Wine**						
*SMOX*	2.10	7.80	3.72	5.62E-07	0.010	Cellular functions including scavenging reactive oxygen species; neurotransmission; oxodpredictase activity; Involved in cytochrome p450 pathways; biological oxidation pathway; and metabolism
*UPK1B*	3.16	0.42	0.13	1.86E-05	0.065	Member of tetraspanin family; structural molecular activity; epithelial cell differentiation
*DTD2*	11.88	5.50	0.46	3.26E-05	0.095	tRNA metabolic process regulation of translation fideltiy; hydrolase activity
*KIAA1715*	56.16	38.18	0.68	7.40E-06	0.050	Blood coagulation; hemostatsis, response to wounding
*C4orf51*	0.36	3.37	9.46	1.31E-05	0.057	Not available
*MTRNR2L9*	3.66	12.26	3.35	8.53E-06	0.050	Neuroprotective and antipoptotic factor
**Liquor**						
*CXCL2*	6.44	1.31	0.20	3.03E-06	0.024	Chemokine activity; immune response; inflammatory response; chemokine signaling; signaling by GPCR
*RBBP7*	43.96	29.27	0.67	2.48E-05	0.097	Cell proliferation; negative regulation of transcritpion from RNA polymerase II promoter; packaging of telomere ends; Development NOTCH1-mediated pathway for NFKB activity
*SLC5A12*	11.17	8.87	0.79	8.95E-07	0.014	Symporter activity; ion transport;
*CACNA1C*	29.31	51.19	1.75	2.44E-05	0.097	Cellular calcium ion homeostasis; cell-to-cell sginaling; regulation of hormone levels such as insulin; mood disorders and biopolar disease

^1^P values are raw p values

^2^Q Value is the smallest FDR at which this gene is called significant.

**Fig 3:**
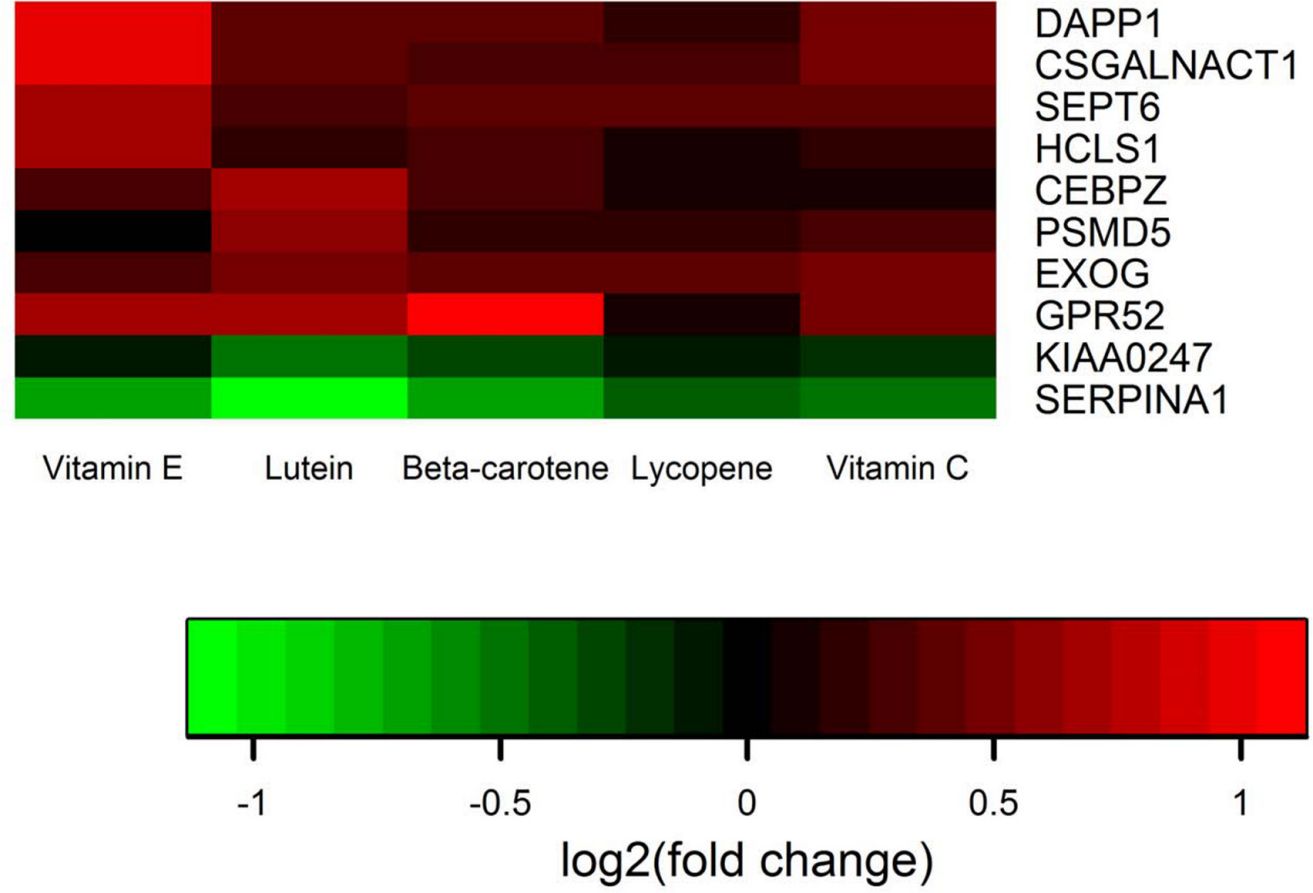
HEATMAP of differentially expressed genes by antioxidants and carotenoids.

Beta carotene (one gene), total alpha tocopherol or Vitamin E (four genes) and lutein+ zeaxanthin (five genes) were associated with differentially expressed genes between high and low nutrient intakes. Vitamin C and lycopene intake were not associated with any differentially expressed genes when the FDR was controlled at <0.1 (Table 4). With few exceptions high intake of antioxidants and carotenoids were associated with higher gene expression than low nutrient intake. Genes differentially expressed by high intake versus low intake of dietary antioxidants and carotenoids are shown in Fig 3. Three genes were significantly differentially expressed based on recent NSAIDs (Table 4), while three genes showed differential expression between high and low oxidative balance score (Table 4). Two of the three genes associated with NSAIDs were up-regulated among users while all of these genes associated with OBS were up-regulated among those with a higher OBS (lower oxidative stress).

**Table 4:** Diet and oxidative balance score associated with differences in gene expression between the upper and lower tertile of expression where the False Discovery Rate (FDR) was set to <0.10.

Gene	Average Adjusted Count, Low Tertile Group	Average Adjusted Count, High Tertile Group	Fold Change	P Value^2^	Q Value^3^	Function/known pathways or disease relationships
**Beta Carotene**						
*GPR52*	4.48	9.46	2.11	3.25E-06	0.057	Cell surface receptor linked signal transduction, G-protein coupled receptor (GPCR) protein signaling pathway
**Lutein/Zeazanthine**						
*PSMD5*	19.37	28.65	1.48	4.27E-05	0.096	Cell Cycle processes; protein binding involved in protein folding; Parkin-Ubiquitin Proteasomal System Pathway
*KIAA0247*	92.37	67.78	0.73	6.12E-05	0.096	Biological Processes
*CEBPZ*	36.19	57.57	1.59	1.38E-05	0.096	Transcription regulation; DNA binding; direct p53 effectors pathway
*EXOG*	15.68	22.09	1.41	4.37E-05	0.096	Endonuclease activity; nucleic acid binding; hydrolase activity
*SERPINA1*	64.53	29.45	0.46	5.76E-05	0.096	Response to hypoxia; acute inflammatory response; defense response; pathways include hemostasis and FOXA1 transcription factor network
**Vitamin E**						
*DAPP1*	8.84	17.73	2.01	2.95E-05	0.097	Protein amino acid dephosphorylation;
*SEPT6*	29.29	48.49	1.66	1.03E-05	0.097	Cytokinesis, cell cycle; cell division; required for normal organization of the actin cytoskeleton; associated with acute myeloid leukemia; pathway include bacterial invasion of epithelial cells
*CSGALNACT1*	6.65	13.28	2.00	2.92E-05	0.097	Metabolic processes; glucuronsyltransferase activity
*HCLS1*	29.73	47.28	1.59	1.78E-05	0.097	Protein kinase binding; antigen receptor-coupled tyrosine kinase; response to hormones; pathways include immune response Fc epsilon RI pathway
**NSAIDS**						
*TMCO6*	9.89	18.47	1.87	1.81E-05	0.09	Protein transporter activity; intracellular protein transport
*ST8SIA4*	11.59	6.25	0.54	4.47E-06	0.05	Cellular protein metabolic process; nervous system development protein linked glycosylation
*STEAP3*	14.53	22.99	1.58	2.51E-05	0.09	Metal ion binding; apoptotic process; cell cycle; TP53 signaling pathway
**Oxidative Balance Score**						
*SYCP3*	16.27	28.24	1.74	1.48E-06	0.026	Cell Cycle Process; cell division; DNA binding
*HDX*	2.77	5.08	1.84	1.56E-05	0.091	Regulation of transcription; DNA binding
*NRG4*	5.61	11.27	2.01	8.55E-06	0.075	Growth factor activity

^1^No differentially expressed genes with FDR <0.1 identified for Vitamin C and lycopene

^2^P values are raw p values

^3^Q Value is the smallest FDR at which this gene is called significant.

## Discussion

We observed that several genes were differentially expressed between high and low categories of diet and lifestyle variables. Genes that were differentially expressed among recent cigarette smokers and alcohol consumers were usually down-regulated. Likewise, individuals with greater intakes of antioxidants and carotenoids were more likely to have down-regulated genes compared to low intakes of these nutrients. We observed that specific type of alcohol consumed influenced specific genes, with virtually no overlap between differentially expressed genes by alcohol type, suggesting that different types of alcohol have specific biological actions beyond that of alcohol in general.

In assessing the data, it is important to keep in mind several features of gene expression data. First, gene expression profile is relevant to current exposure of diet and lifestyle variables. Thus, we assessed current smokers, recent consumers of alcohol, current NSAID users, and diet close to the time of diagnosis, when the tissue would have been biopsied. Thus this represents gene expression at the time of diagnosis. For some variables such as NSAID or specific dietary factors, recent use may have been too far removed to accurately correlate with gene expression. This could explain why few gene were de-regulated for NSAIDs specifically or for dietary antioxidants and alcohol. While lack of findings could indicate too dissonant timing between exposure and tissue sample ascertainment, finding associations would imply that the exposure is recent enough to alter the expression. It is also important to note that the data being analyzed are gene expression data and does not reflect protein expression. Additionally, we have utilized colonic non-tumor tissue, so genes would have to be expressed in colon tissue for detection. Thus, these diet and lifestyle factors could influence other genes in other tissue sources. Utilizing colonic tissue that is located close to a tumor could alter gene expression, however, it would alter it is a manner that was not dependent on diet or lifestyle exposure.

We focused on networks where several of the genes in our data were de-regulated based on lifestyle factors that could result in oxidative imbalance or stress. We utilized IPA to identify networks and upstream regulators to gain insight into biological mechanisms associated with the observed gene expression changes for recent cigarette smoking since that exposure was associated with enough de-regulated genes to assess networks. To control for type I errors we set the estimated FDR to 0.1 to generate more complete networks. Causal networks in IPA are based on the Ingenuity Knowledge Base, which is contains information from the literature as well as from other databases [29]. IPA defines upstream regulators as any gene or small molecular that has been observed to affect gene expression, either directly or indirectly [29]. Up-stream regulators are defined as those that are likely connected to our dataset genes through a set of direct or indirect relationships. The causal network analysis in IPA builds on the upstream regulator analysis and builds a more comprehensive picture of possible root causes linked to the gene expression profiles. Causal network analysis includes intermediaries which are intermediate regulators that link de-regulated genes to upstream regulators. In our data, de-regulated genes were generally linked to the upstream regulator through intermediaries. While there were several networks in which genes in our data were involved, we focused on those networks that contained several genes that were importantly associated with the upstream regulators.

Recent cigarette smoking was the only lifestyle factor evaluated that had a sufficient number of genes de-regulated to evaluate in IPA. In our data, genes de-regulated by recent cigarette smoking were linked to causal networks involving *TEK* (endothelial tyrosine kinase also known as *TIE2*), *MAP2K3*, and *PNBP4*. The literature supports up-regulation of these pathways by cigarette smoking [30–32]. The angiogenic growth factors ANG1 and ANG2 act through TEK. It is involved in vascular integrity and is mainly expressed in endothelial cells. TEK has been shown to be down-regulated among smokers and individuals with chronic obstructive pulmonary disease [30]. Using IPA and the upregulated genes identified with an FDR of <0.1 the TEK network was significantly enriched and TEK was predicted to be up-regulated. However in our data, current smokers had lower levels of TEK expression than never smokers (0.55 fold change; unadjusted p value of 0.046) as previously reported. Since *TEK* was not significant using an FDR <0.1 it was not uploaded into IPA or identified as significantly differentially expressed. It is likely that other intervening features mediated the predicted and observed association. Few studies have evaluated smoking and gene expression. One study conducted by Nielsen and colleagues studied 28 individuals with Crohn’s disease who smoked and 14 who did not [33]. They observed three differentially expressed genes after replication by RT PCR, *RNF138*, *MT2A*, and *STEAP3*. While these genes were not differentially expressed in our sample of individuals without Crohn’s Disease, we did observe that STEAP3 was associated with recent aspirin/NSAID use. Oxidative stress can lead to activation of several MAPK signaling cascades [34]. MAP2K3 is activated by cytokines and environmental stress and is involved in regulation of p38. MAPK pathways have been linked to cigarette smoking [35]. Our data support the literature that suggest MAPK signaling being influenced by tobacco given the number of up-regulated genes in the MAP2K3 network. Many of the genes in the TEK and MAP2K3 network were also central to the PNBP4 network. Given the heavy overlap in number of enriched genes in these networks supports a strong role of cigarette smoking influencing MAPK signaling and inflammation-related genes.

Few genes differentially expressed by overall alcohol consumption and by subtype. Only one gene was differentially expressed in more than one category; CACNA1C was up-regulated among those who consumed high levels of liquor as well as those who had high overall alcohol levels. Disrupted genes varied by type of alcohol consumed, however there were similarities in function for specific types of alcohol. For instance, two of the five genes de-regulated for alcohol were involved in biological mechanisms of calcium and two were involved in MAPK signaling. Similarly, two of the six de-regulated genes influenced by beer consumption were involved in *AKT* signaling, two were involved in immune response and inflammation and two had functions related to apoptosis. Two of the five genes de-regulated by wine consumption were associated with biological oxidation pathway and response to wound healing as was one of the four deregulated genes associated with liquor consumption.

Previous studies have shown that diet modification can alter gene expression [20]. In controlled setting, beta carotene has been shown to exhibit anti-inflammatory properties by inhibiting NFκB activation [36]. Lutein, a carotenoid with anti-inflammatory properties, has previously been shown to influence cell cycle progression; cells treated with lutein showed increased expression of *IGF1R*, *EGFR*, *BRCA1*, *CDK5*, *KLK14*, and *PCA3* and decreased expression of *GSTP1* and *RASSF1* [37]. However, few of the dietary antioxidants and carotenoids we evaluated altered gene expression and did not alter expression of those previously reported. We evaluated over 17,000, and restricted those that were considered differentially expressed based on FDR. Other studies of few genes did not have make these adjustments. In our data lutein+zeaxanthin altered five genes, vitamin E altered expression of four genes, and beta carotene altered expression of one gene. The genes that were altered were unique by antioxidants and carotenoids and were involved in biologically relevant functions including cell cycle processes, transcription factors response to hypoxia, inflammation, metabolic and apoptotic processes.

We also assessed the OBS, a composite of the individual variables assessed and only observed altered expression in three genes which were associated with cell cycle process, transcription and growth factor activity. The OBS has previously been associated with reduced risk of colon cancer [17].

We used RNAseq to determine differential gene expression by lifestyle factor given its wide-spread use, high level of repeatability, the cost, and amount of sample required to generate the data. As RNAseq produces global gene expression data for each RNA sample, it is an ideal method to undertake a discovery study as we have done here [38,39]. However, it is recognized that different platforms carry different technical weaknesses that can effect cross platform replication such as library construction methods and number of amplification cycles required for data generation. Comparative studies estimate that around 50% of results may replicate between platforms [40,41]. Given the number of associations identified and the limited amount of sample available from the FFPE tissue, it is not reasonable or feasible to replicate all findings. To obtain a crude estimate of replication by qPCR methods, we analyzed data from two markers, HELZ and DUT. All results showed the same direction of differential expression as observed with RNAseq, however the magnitude and level of significance varied by test. DUT was significantly associated with smoking (p = 0.01), HELZ was of marginal significance with alcohol (p = 0.08), and although smokers had lower HELZ expression than never smokers as with RNAseq, the difference was not statistically significant (p = 0.34). Thus, it is essential to validate these findings in other populations using the methods we used here as well as other platforms to better understand associations between lifestyle factors and gene expression.

Our data support the hypothesis that levels of diet and lifestyle factors associated with oxidative balance and stress alter gene expression profiles. Cigarette smoking had the most influence on expression levels. Many of the pathways and networks associated with de-regulated genes involved cell cycle and oxidative stress. Also of interest is the observation that different types of alcohol affected expression of different genes. Confirmation of these findings by other similarly designed studies is needed.

## Supporting Information

S1 Table Summary of genes showing differential expression at the FDR level of 0.1 with recent cigarette smoking. (DOCX)

## References

[1] Slattery ML, Curtin K, Anderson K, Ma KN, Ballard L, Edwards S, et al (2000) Associations between cigarette smoking, lifestyle factors, and microsatellite instability in colon tumors. J Natl Cancer Inst 92: 1831–1836. 11078760 10.1093/jnci/92.22.1831

[2] Slattery ML, Potter JD, Samowitz W, Bigler J, Caan B, Leppert M (1998) NAT2, GSTM-1, cigarette smoking, and risk of colon cancer. Cancer Epidemiol Biomarkers Prev 7: 1079–1084. 9865425

[3] Giovannucci E, Colditz GA, Stampfer MJ, Hunter D, Rosner BA, Willett WC, et al (1994) A prospective study of cigarette smoking and risk of colorectal adenoma and colorectal cancer in U.S. women. Journal of the National Cancer Institute 86: 192–199. 8283491 10.1093/jnci/86.3.192

[4] Giovannucci E, Rimm EB, Stampfer MJ, Colditz GA, Ascherio A, Kearney J, et al (1994) A prospective study of cigarette smoking and risk of colorectal adenoma and colorectal cancer in U.S. men. Journal of the National Cancer Institute 86: 183–191. 8283490 10.1093/jnci/86.3.183

[5] Giovannucci E (2001) An updated review of the epidemiological evidence that cigarette smoking increases risk of colorectal cancer. Cancer Epidemiol Biomarkers Prev 10: 725–731. 11440957

[6] Chao A, Thun MJ, Jacobs EJ, Henley SJ, Rodriguez C, Calle EE (2000) Cigarette smoking and colorectal cancer mortality in the cancer prevention study II. J Natl Cancer Inst 92: 1888–1896. 11106680 10.1093/jnci/92.23.1888

[7] Talukder MA, Johnson WM, Varadharaj S, Lian J, Kearns PN, El-Mahdy MA, et al (2011) Chronic cigarette smoking causes hypertension, increased oxidative stress, impaired NO bioavailability, endothelial dysfunction, and cardiac remodeling in mice. Am J Physiol Heart Circ Physiol 300: H388–396. 10.1152/ajpheart.00868.2010 21057039 PMC3023256

[8] Chavez J, Cano C, Souki A, Bermudez V, Medina M, Ciszek A, et al (2007) Effect of cigarette smoking on the oxidant/antioxidant balance in healthy subjects. Am J Ther 14: 189–193. 17414589 10.1097/01.psp.0000249918.19016.f6

[9] Mena S, Ortega A, Estrela JM (2009) Oxidative stress in environmental-induced carcinogenesis. Mutat Res 674: 36–44. 10.1016/j.mrgentox.2008.09.017 18977455

[10] Petersen DR (2005) Alcohol, iron-associated oxidative stress, and cancer. Alcohol 35: 243–249. 16054986 10.1016/j.alcohol.2005.03.013

[11] Nagamma T, Bhutia RD, Pokharel DR, Yadav S, Baxi J (2012) Influence of alcohol consumption on oxidative stress and antioxidant status in cancer patients—case-control study from Western Nepal. Asian Pac J Cancer Prev 13: 3513–3517. 22994787 10.7314/apjcp.2012.13.7.3513

[12] Oyesanmi O, Snyder D, Sullivan N, Reston J, Treadwell J, Schoelles KM (2010) Alcohol consumption and cancer risk: understanding possible causal mechanisms for breast and colorectal cancers. Evid Rep Technol Assess (Full Rep): 1–151. PMC4781486 23126574

[13] Slattery ML, Benson J, Curtin K, Ma KN, Schaeffer D, Potter JD (2000) Carotenoids and colon cancer. Am J Clin Nutr 71: 575–582. 10648274 10.1093/ajcn/71.2.575

[14] Slattery ML, Edwards SL, Anderson K, Caan B (1998) Vitamin E and colon cancer: is there an association? Nutr Cancer 30: 201–206. 9631491 10.1080/01635589809514664

[15] Stone WL, Papas AM (1997) Tocopherols and the etiology of colon cancer. J Natl Cancer Inst 89: 1006–1014. 9230882 10.1093/jnci/89.14.1006

[16] Satia-Abouta J, Galanko JA, Martin CF, Potter JD, Ammerman A, Sandler RS (2003) Associations of micronutrients with colon cancer risk in African Americans and whites: results from the North Carolina Colon Cancer Study. Cancer Epidemiol Biomarkers Prev 12: 747–754. 12917206

[17] Slattery ML, Lundgreen A, Welbourn B, Wolff RK, Corcoran C (2012) Oxidative balance and colon and rectal cancer: Interaction of lifestyle factors and genes. Mutation Research 734: 30–40. 10.1016/j.mrfmmm.2012.04.002 22531693 PMC3372651

[18] Goodman M, Bostick RM, Gross M, Thyagarajan B, Dash C, Flanders WD (2010) Combined measure of pro- and anti-oxidant exposures in relation to prostate cancer and colorectal adenoma risk: an update. Annals of epidemiology 20: 955–957. 10.1016/j.annepidem.2010.08.011 21074110 PMC3008422

[19] Goodman M, Bostick RM, Dash C, Terry P, Flanders WD, Mandel J (2008) A summary measure of pro- and anti-oxidant exposures and risk of incident, sporadic, colorectal adenomas. Cancer causes & control: CCC 19: 1051–1064. 10.1007/s10552-008-9169-y 18543072

[20] Ornish D, Magbanua MJ, Weidner G, Weinberg V, Kemp C, Green C, et al (2008) Changes in prostate gene expression in men undergoing an intensive nutrition and lifestyle intervention. Proc Natl Acad Sci U S A 105: 8369–8374. 10.1073/pnas.0803080105 18559852 PMC2430265

[21] Morel Y, Barouki R (1999) Repression of gene expression by oxidative stress. Biochem J 342 Pt 3: 481–496. 10477257 PMC1220487

[22] Slattery ML, Edwards SL, Palmer L, Curtin K, Morse J, Anderson K, et al (2000) Use of archival tissue in epidemiologic studies: collection procedures and assessment of potential sources of bias. Mutat Res 432: 7–14. 10729707 10.1016/s1383-5726(99)00010-2

[23] Samowitz WS, Curtin K, Lin HH, Robertson MA, Schaffer D, Nichols M, et al (2001) The colon cancer burden of genetically defined hereditary nonpolyposis colon cancer. Gastroenterology 121: 830–838. 11606497 10.1053/gast.2001.27996

[24] Edwards S, Slattery ML, Mori M, Berry TD, Caan BJ, Palmer P, et al (1994) Objective system for interviewer performance evaluation for use in epidemiologic studies. Am J Epidemiol 140: 1020–1028. 7985650 10.1093/oxfordjournals.aje.a117192

[25] Liu K, Slattery M, Jacobs D Jr, Cutter G, McDonald A, Van Horn L, et al (1994) A study of the reliability and comparative validity of the cardia dietary history. Ethn Dis 4: 15–27. 7742729

[26] Benjamini Y, Hochberg Y (1995) Controlling the false discovery rate: a practical and powerful approach to multiple testing. Journal of the Royal Statistical Society 57: 289–300.

[27] Love MI, Huber W, Anders S (2014) Moderated estimation of fold change and dispersion for RNA-seq data with DESeq2. Genome Biol 15: 550 25516281 10.1186/s13059-014-0550-8 PMC4302049

[28] (2014) QIAGEN's Ingenuity Pathway Analysis

[29] Kramer A, Green J, Pollard J Jr, Tugendreich S (2014) Causal analysis approaches in Ingenuity Pathway Analysis. Bioinformatics 30: 523–530. 10.1093/bioinformatics/btt703 24336805 PMC3928520

[30] Llinas L, Peinado VI, Ramon Goni J, Rabinovich R, Pizarro S, Rodriguez-Roisin R, et al (2011) Similar gene expression profiles in smokers and patients with moderate COPD. Pulm Pharmacol Ther 24: 32–41. 10.1016/j.pupt.2010.10.010 20970515

[31] Mossman BT, Lounsbury KM, Reddy SP (2006) Oxidants and signaling by mitogen-activated protein kinases in lung epithelium. Am J Respir Cell Mol Biol 34: 666–669. 16484683 10.1165/rcmb.2006-0047SF PMC2644227

[32] Arredondo J, Chernyavsky AI, Jolkovsky DL, Pinkerton KE, Grando SA (2007) Receptor-mediated tobacco toxicity: alterations of the NF-kappaB expression and activity downstream of alpha7 nicotinic receptor in oral keratinocytes. Life Sci 80: 2191–2194. 17291542 10.1016/j.lfs.2007.01.013 PMC1973165

[33] Nielsen OH, Bjerrum JT, Csillag C, Nielsen FC, Olsen J (2009) Influence of smoking on colonic gene expression profile in Crohn's disease. PLoS One 4: e6210 10.1371/journal.pone.0006210 19603079 PMC2708910

[34] Arredondo J, Chernyavsky AI, Jolkovsky DL, Pinkerton KE, Grando SA (2008) Receptor-mediated tobacco toxicity: acceleration of sequential expression of alpha5 and alpha7 nicotinic receptor subunits in oral keratinocytes exposed to cigarette smoke. FASEB J 22: 1356–1368. 10.1096/fj.07-9965.com 18450646

[35] He B, Luo B, Chen Q, Zhang L (2013) Cigarette smoke extract induces the expression of GRP78 in A549 cells via the p38/MAPK pathway. Mol Med Rep 8: 1683–1688. 10.3892/mmr.2013.1724 24126384

[36] Bai SK, Lee SJ, Na HJ, Ha KS, Han JA, Lee H, et al (2005) beta-Carotene inhibits inflammatory gene expression in lipopolysaccharide-stimulated macrophages by suppressing redox-based NF-kappaB activation. Exp Mol Med 37: 323–334. 16155409 10.1038/emm.2005.42

[37] Rafi MM, Kanakasabai S, Gokarn SV, Krueger EG, Bright JJ (2014) Dietary Lutein Modulates Growth and Survival Genes in Prostate Cancer Cells. J Med Food. 10.1089/jmf.2014.0003 25162762

[38] Dorr C, Wu B, Guan W, Muthusamy A, Sanghavi K, Schladt DP, et al (2015) Differentially expressed gene transcripts using RNA sequencing from the blood of immunosuppressed kidney allograft recipients. PLoS One 10: e0125045 10.1371/journal.pone.0125045 25946140 PMC4422721

[39] Guo Y, Sheng Q, Li J, Ye F, Samuels DC, Shyr Y (2013) Large scale comparison of gene expression levels by microarrays and RNAseq using TCGA data. PLoS One 8: e71462 10.1371/journal.pone.0071462 23977046 PMC3748065

[40] Mestdagh P, Hartmann N, Baeriswyl L, Andreasen D, Bernard N, Chen C, et al (2014) Evaluation of quantitative miRNA expression platforms in the microRNA quality control (miRQC) study. Nat Methods 11: 809–815. 10.1038/nmeth.3014 24973947

[41] Fonseca NA, Marioni J, Brazma A (2014) RNA-Seq gene profiling—a systematic empirical comparison. PLoS One 9: e107026 10.1371/journal.pone.0107026 25268973 PMC4182317

