# Supplementary material for: Differential Gene Expression in Colon Tissue Associated With Diet, Lifestyle, and Related Oxidative Stress
Source: PLoS One. 2015 Jul 31;10(7):e0134406. doi: 10.1371/journal.pone.0134406 (PMC4521956; doi:10.1371/journal.pone.0134406)
Supplement: S1 Table — (DOCX) [file pone.0134406.s001.docx]

| S1 Table. Summary of genes showing differential expression at the FDR level of 0.1 with recent cigarette smoking | | | | | | | |
| --- | --- | --- | --- | --- | --- | --- | --- |
| Gene Name | | | Average Adjusted Count, Never Smoker | Average Adjusted Count, Current Smoker | Fold Change | P Value | Q Value |
| *TAC1* | Tachykinin1 | | 0.33 | 2.25 | 6.77 | 1.79E-04 | 0.079 |
| *PKP1* | Plakophilin 1 | | 1.09 | 4.82 | 4.44 | 2.27E-04 | 0.086 |
| *C20orf26* | Chromosome 20 open reading frame 26 | | 0.45 | 2.57 | 5.76 | 3.76E-05 | 0.044 |
| *LYZ* | Lysozyme | | 38.99 | 90.23 | 2.31 | 4.03E-05 | 0.044 |
| *LAMB4* | Laminin, Beta 4 | | 1.07 | 3.54 | 3.32 | 1.80E-04 | 0.079 |
| *UPB1* | Beta-ureidopropionase | | 1.61 | 5.11 | 3.17 | 1.16E-04 | 0.068 |
| *VSIG1* | V-set and immunoglobulin domains-containing protein 1 | | 0.63 | 3.39 | 5.36 | 2.22E-04 | 0.086 |
| *PEX5L* | Peroxisome biogenesis factor 5-like | | 1.44 | 9.86 | 6.87 | 1.62E-05 | 0.024 |
| *CD207* | CD207 Antigen | | 0.42 | 2.53 | 6.04 | 5.47E-05 | 0.047 |
| *TECTB* | Tectorin Beta | | 0.73 | 2.69 | 3.68 | 1.51E-04 | 0.073 |
| *CHRNA2* | Cholinergic receptor, neuronal nicotinic, alpha polypeptide 2 | | 0.53 | 2.11 | 3.97 | 7.46E-05 | 0.057 |
| *C1orf61* | Chromosome 1 open reading frame 61 | | 1.46 | 8.36 | 5.73 | 3.72E-05 | 0.044 |
| *CRYGN* | Crystallin, Gamma-N | | 0.30 | 2.94 | 9.85 | 1.15E-04 | 0.068 |
| *TRPV5* | Transient receptor potential cation channel, subfamily V, Member 5 | | 0.16 | 1.69 | 10.91 | 5.65E-05 | 0.047 |
| *DUT* | dUTP pyrophosphatase | | 12.79 | 4.26 | 0.33 | 7.88E-07 | 0.003 |
| *CDH15* | Cadherin 15 | | 0.76 | 3.56 | 4.70 | 1.20E-04 | 0.068 |
| *SAG* | S-Antigen | | 0.84 | 4.73 | 5.60 | 1.10E-05 | 0.019 |
| *SLC6A11* | Solute carrier family 6, Member 11 | | 0.69 | 2.20 | 3.20 | 2.67E-05 | 0.036 |
| *TRPM1* | Transient receptor potential cation channel, subfamily M, member 1 | | 0.61 | 4.11 | 6.76 | 1.03E-04 | 0.067 |
| *LHCGR* | Luteinizing hormone/choriogonadotropin receptor | | 1.67 | 8.83 | 5.29 | 1.01E-05 | 0.019 |
| *SIX3* | Sine Oculis Homeobox, Drosophila, Homolog of , 3 | | 0.20 | 4.27 | 21.49 | 8.10E-06 | 0.018 |
| *CHRNA1* | Cholinergic receptor, neuronal nicotinic, alpha polypeptide 1 | | 0.87 | 3.22 | 3.68 | 1.40E-04 | 0.070 |
| *CACNG8* | Calcium channel, voltage-dependent, gamma-8 subunit | | 1.67 | 9.18 | 5.49 | 8.38E-05 | 0.061 |
| *FBN3* | Fibrillin 3 | | 1.07 | 5.03 | 4.70 | 1.90E-04 | 0.079 |
| *DLX1* | Distal-less homeobox 1 | | 0.33 | 1.91 | 5.86 | 7.48E-07 | 0.003 |
| *PRSS35* | Protease, serine, 35 | | 3.86 | 1.86 | 0.48 | 1.89E-04 | 0.079 |
| *SLC5A12* | Solute carrier family 5 (sodium/glucose cotransporter), member 12 | | 6.41 | 27.05 | 4.22 | 5.80E-08 | 0.001 |
| *ADCY8* | Adenylate cyclase 8 | | 0.11 | 1.78 | 16.90 | 1.52E-05 | 0.024 |
| *NLRP4* | NLR Family, PYRIN Domain-Containing 4 | | 0.17 | 2.90 | 16.65 | 4.89E-06 | 0.014 |
| *HFM1* | ATP-dependent DNA Helicase, S Cerevisiae, Homolog of | | 0.42 | 3.71 | 8.84 | 5.77E-06 | 0.014 |
| *NKX6-1* | NK6, Drosophila, Homolog of, 1 | | 0.05 | 1.74 | 32.79 | 1.26E-04 | 0.068 |
| *GSG1L* | GSG1-like | | 1.76 | 4.21 | 2.39 | 9.85E-05 | 0.066 |
| *FGA* | Fibrinogen, A Alpha Polypeptide | | 0.16 | 4.38 | 28.01 | 6.30E-07 | 0.003 |
| *WDR87* | WD Repeat-containing protein 87 | | 1.04 | 6.43 | 6.18 | 4.55E-05 | 0.044 |
| *GOLGA6L2* | Golgin A6 family-like 2 | | 2.66 | 2.52 | 0.95 | 2.25E-04 | 0.086 |
| *C11orf16* | Chromosome 11 open reading frame 16 | | 0.32 | 2.05 | 6.52 | 5.90E-05 | 0.047 |
| *TMEM78* | Transmembrane protein 78 | | 0.61 | 1.69 | 2.75 | 1.55E-04 | 0.073 |
| *TMIE* | Transmembrane inner ear-expressed gene | | 0.27 | 2.32 | 8.65 | 1.36E-04 | 0.070 |
| *LYPD4* | Ly6/PLAUR domain containing 4 | | 0.07 | 2.27 | 34.11 | 5.09E-05 | 0.047 |
| *S100A7A* | S100 Calcium-binding protein A7 | | 0.43 | 2.47 | 5.77 | 1.58E-04 | 0.073 |
| *HELZ* | Helicase with zinc finger domain | | 10784.43 | 1728.82 | 0.16 | 4.30E-05 | 0.044 |
| *RBM20* | Cardiomyopathy, dilated, 1DD | | 2.74 | 8.19 | 2.99 | 1.13E-04 | 0.068 |
| *ERVMER34-1* | Endogenous retrovirus group MER34, member 1 | | 1.06 | 3.90 | 3.68 | 9.54E-05 | 0.066 |
| *LTA* | Lymphotoxin-alpha | | 0.81 | 3.86 | 4.77 | 2.22E-04 | 0.086 |
| *YY2* | Transcription Factor YY2 | | 1.64 | 5.48 | 3.34 | 1.39E-04 | 0.070 |
| *CTC-241N9.1* | Uncharacterized Loci | | 0.59 | 2.84 | 4.79 | 3.54E-06 | 0.012 |
|  |  | |  |  |  |  |  |
|  | | P values are raw p values | | |  |  |  |
|  | | Q Value is the smallest FDR at which this gene is called significant. | | | | | |
